# Composition Design and Solidification Mechanism Analysis of Controlled Low-Strength Materials Using Stabilized Stainless Steel Mud

**DOI:** 10.3390/ma19143083

**Published:** 2026-07-17

**Authors:** Zongting Xie, Mingkai Zhou, Peng Gao, Yuqiang Wang, Yuhao Zhou

**Affiliations:** 1 School of Material Science and Engineering, Wuhan University of Technology, Wuhan 430070, China; 18827553974@163.com (Z.X.);; 2 Institute of Wuhan University of Technology, Changzhi 046000, China; 3 Shanxi Coal Measures Solid Waste Building Materials Resource Utilization Technology Innovation Center, Changzhi 046000, China; 4 Shanxi Construction Investment Building Industry Co., Ltd., Taiyuan 030002, China

**Keywords:** stainless steel slag mud, controlled low-strength material, ground granulated blast furnace slag, solidification mechanism, resource utilization

## Abstract

To address the problems of high moisture content, fine particle size, and limited conventional reutilization of stainless steel slag mud (SSSM), controlled low-strength material (CLSM) was prepared using SSSM as the primary solid component and cement together with ground granulated blast furnace slag (GGBS) as cementitious materials. The effects of GGBS replacing cement and SSSM, respectively, on the properties of CLSM and their variation patterns were investigated. Its solidification mechanism was analyzed through simulated control tests, X-ray diffraction (XRD), thermogravimetric–differential thermogravimetric analysis (TG-DTG), and scanning electron microscopy (SEM). The results show that, when GGBS replaces cement, the water-to-solid ratio and bleeding rate increase, while the compressive strength at all curing ages decreases overall; however, the 28 d strength still meets the requirement for CLSM. When GGBS replaces SSSM, the water-to-solid ratio and bleeding rate increase with GGBS fraction, and the compressive strength at all curing ages increases overall. At a GGBS fraction of 18%, the water-to-solid ratio reaches 0.363, the bleeding rate reaches 5%, and the 28 d and 60 d compressive strengths reach 7.3 and 11.2 MPa, respectively, representing increases of 23.3 and 21.4 times compared with the system without GGBS (0.3 and 0.5 MPa). The simulated SSSM substitution tests show that a synergistic solidification effect exists between SSSM and GGBS and contributes to strength development. Microstructural analysis shows that GGBS undergoes hydration under the alkali–sulfate environment provided by SSSM, generating ettringite (AFt) and calcium silicate hydrate (C-S-H gel), which fill pores and thereby enhance strength, while calcium hydroxide (Ca(OH)_2_) provides an alkaline environment and promotes the participation of potentially active components in SSSM in the synergistic solidification process.

## 1. Introduction

Stainless steel slag contains valuable metals such as chromium, nickel, and iron. The main approach for resource utilization of stainless steel slag is to extract and recover these valuable metals through wet grinding. However, this process simultaneously generates a large amount of stainless steel slag tailings. Due to their high fineness and high water fraction, these tailings are also referred to as stainless steel slag mud (SSSM). The annual production of stainless steel slag in China is substantial, yet its resource utilization remains insufficient. Landfilling and stockpiling disposal not only occupy land resources but also cause environmental pollution. Therefore, it is necessary to develop large-scale resource utilization pathways for SSSM.

Existing studies on the resource utilization of stainless steel slag have mainly focused on two pathways: cement admixture and concrete aggregate. In the field of cement admixtures, stainless steel slag possesses a certain degree of cementitious activity. For example, Chen et al. [1] systematically compared dry- and wet-processed AOD slag and found that dry-processed slag exhibited better cementitious activity, with a 28 d compressive strength activity index of 53.45% when replacing 30% cement. Ghorbani et al. [2] investigated alkali-activated electric arc furnace stainless steel slag (EAFSS) with GGBS blends, and reported that up to 50% EAFSS replacement still achieved a 28 d compressive strength exceeding 85 MPa, with chromium immobilized in a stable spinel phase showing negligible leaching risk. However, compared with raw stainless steel slag, SSSM has a high moisture content of up to 15% and tends to self-harden and agglomerate. It requires drying and grinding before being used as an admixture, resulting in high energy consumption. Moreover, the current national standard General Purpose Portland Cement (GB 175-2023) [3] no longer lists steel slag as an allowed main admixture for general-purpose cement. Consequently, the application of SSSM in cement production is restricted. Even if pretreatment is completed, SSSM still cannot be introduced into general-purpose cement production lines, substantially limiting its resource utilization routes in the cement industry. Recent studies on the activation of raw stainless steel slag have also confirmed that [4] the energy cost associated with drying pretreatment is difficult to reduce effectively, and the constraints of the national standard remain unavoidable.

In the direction of aggregate utilization, Wei et al. [5] investigated the recycling of steel slag as cementitious materials and fine aggregate in concrete, reporting that 20% steel slag powder increased compressive strength by 34.57%, with AFt and C-S-H gel identified as the main hydration products. Zheng and Deng [6] studied geopolymer concrete with fully recycled aggregates based on mixed blast furnace slag and stainless steel slag. Huang et al. [7] prepared road base materials using stainless steel AOD slag, fly ash, and recycled aggregates, and found that the incorporation of AOD slag effectively enhanced the hydration and pozzolanic reactions, leading to improved mechanical strength. All of the aforementioned studies were conducted on raw stainless steel slag with coarse particles and well-graded particle size distribution. In contrast, SSSM loses its original gradation during the grinding process, and its fineness is comparable to that of steel slag powder, making it difficult to serve as an aggregate. Therefore, the aggregate-based utilization strategy developed for raw stainless steel slag cannot be directly transferred to SSSM. A few studies [8] have attempted to apply SSSM in cement-stabilized base materials for road construction. However, the high fines content of SSSM leads to defects such as significant drying shrinkage and strength degradation, restricting its usage only to low-load subgrade layers and preventing its widespread use in large-scale backfill engineering.

Controlled low-strength material (CLSM) is a self-leveling, low-strength backfill material prepared from a mixture of binder, cementitious materials, and water [9]. Compared with traditional backfill materials, CLSM offers strong adaptability to raw material characteristics, requiring only that the slurry achieves self-leveling and that the compressive strength is adjustable within the range of 0.5–8.3 MPa [10,11,12,13,14,15,16]. The relatively flexible performance requirements broaden the range of available raw materials, allowing greater tolerance for variations in particle size, moisture content, and chemical composition of industrial solid wastes. Previous studies have demonstrated that CLSM can effectively utilize various industrial and municipal solid wastes, including steel slag, ground granulated blast furnace slag (GGBS), circulating fluidized bed fly ash, municipal solid waste incineration ash, and dewatered sludge, all of which have been incorporated into CLSM formulations [17,18,19,20,21]. In recent years, the use of various solid wastes as substitutes for cement or sand in CLSM production has become a research hotspot. In comparison, research on the application of stainless steel slag, particularly SSSM, in CLSM remains rather limited. Given its high moisture content, fine particle size, and potential reactivity, incorporating SSSM into the CLSM raw material system may alleviate the constraint imposed by its high moisture content on resource utilization, while taking advantage of its fineness and latent reactivity. Therefore, this approach could represent a promising pathway for the low-cost resource utilization of SSSM.

Overall, systematic research dedicated to the resource utilization of SSSM remains limited. Most backfill studies focusing on stainless steel metallurgical slag adopt dried raw slag, lacking low-energy technical routes to directly utilize as-received SSSM without energy-intensive drying pretreatment. The solidification interaction between SSSM and cementitious components is only qualitatively characterized, and the coupled contributions of physical filling and chemical activation derived from ultra-fine SSSM have not been quantitatively decoupled. Furthermore, existing CLSM systems based on stainless steel slag and GGBS generally incorporate Portland cement; a fully solid-waste, cement-free backfill system dominated by as-received SSSM with full cement substitution by GGBS has not been reported. Given the inherent high-moisture, ultra-fine characteristics of SSSM and the broad raw material tolerance of CLSM, producing cement-free CLSM with untreated raw SSSM offers a promising low-cost pathway for the large-scale resource utilization of SSSM.

Unlike previous studies that used steel slag blended with cement for CLSM preparation, this work directly utilizes SSSM to construct a cement-free CLSM system with GGBS replacing cement. To address the aforementioned limitations, a two-stage mix proportion experiment is designed to investigate the evolution of the water-to-solid ratio, bleeding rate, and compressive strength at various ages when GGBS replaces cement and SSSM, respectively. A simulated substitution control test is designed to quantitatively distinguish the physical filling effect from the chemical activation contribution of SSSM. Furthermore, hydration product characterization and microstructural observations via XRD, TG-DTG, and SEM are integrated to elaborate the solidification mechanism of the GGBS-SSSM composite system, which provides experimental support for the resource utilization of SSSM.

## 2. Materials and Methods

### 2.1. Materials

**Figure 1:**
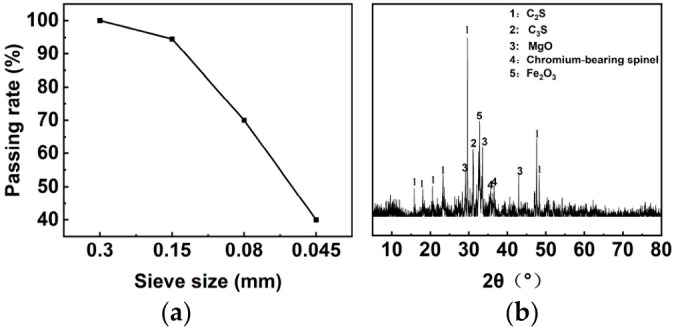
Characterization of SSSM. (**a**) Particle size distribution of SSSM; (**b**) XRD pattern of SSSM.

The SSSM used in this test was sampled from a stainless steel plant in Taiyuan, Shanxi Province. After wet grinding, the SSSM appears as a wet powder with a moisture content of approximately 15%. Figure 1a shows the sieve grading of the SSSM, with a 0.15 mm passing rate of 94.5%. The chemical and mineral compositions of SSSM are presented in Table 1 and Figure 1b, respectively. As shown in Table 1, the main chemical components of SSSM are CaO, SiO_2_, Fe_2_O_3_, MgO, and Al_2_O_3_, accounting for 89.42% of the total mass. Combined with Figure 1b, the mineral composition of SSSM is identified as dicalcium silicate, along with small amounts of tricalcium silicate, periclase, and chromium-containing spinel phases, indicating a certain degree of cementitious activity. According to the Steel Slag Powder Used for Cement and Concrete (GB/T 20491-2017) [22], the 28 d strength activity index of SSSM is 67.7%, and the fraction of free CaO (f-CaO), Ca(OH)_2_, and SO_3_ are 0.05, 0.81, and 0.99 wt.%, respectively. The activity index was determined following the test procedure specified in this standard, where standard mortar specimens were prepared with 30% SSSM replacement of cement. The heavy metal leaching concentrations of SSSM were tested in accordance with Identification Standards for Hazardous Wastes—Identification of Leaching Toxicity (GB 5085.3-2007) [23], and the test results are summarized in Table 2. All detected heavy metal concentrations were lower than the threshold limits for Class I general industrial solid waste specified in the standard, which verifies that the SSSM adopted in this research meets environmental safety requirements.

The GGBS used in this test was obtained from a steelmaking enterprise in Shanxi Province. According to the Ground Granulated Blast Furnace Slag Used for Cement, Mortar and Concrete (GB/T 18046-2017) [24], the specific surface area of the GGBS is 460 m^2^/kg, and its SO_3_ fraction is 2.07 wt.%.

The P·O 42.5 ordinary Portland cement (hereinafter referred to as cement) used in this test was obtained from a cement plant in Changzhi, Shanxi Province. Its initial setting time is 214 min, and final setting time is 339 min. The compressive strengths at 3 d, 7 d, and 28 d are 23.1, 35.4, and 54.7 MPa, respectively. The chemical composition of the cement is shown in Table 1.

### 2.2. Test Scheme Design

A two-stage experimental program was designed to investigate the effect of GGBS fraction on the performance of the SSSM-based CLSM, with cement and GGBS as cementitious materials. In Stage I, GGBS replaced cement at equal mass ratios (3% increments, up to 18%) to study the cement-GGBS composite cementitious system. In Stage II, based on the mix proportion selected from Stage I, GGBS replaced SSSM at equal mass ratios (3% increments, up to 18%) to investigate the effect of GGBS fraction on the performance and solidification efficiency of the SSSM–GGBS CLSM system. The maximum replacement level of 18% was determined based on preliminary tests, which indicated that beyond this level, maintaining the target flowability of 220 mm required excessive mixing water, resulting in noticeable bleeding and segregation. This range is also consistent with previous studies [20] on CLSM using metallurgical slags as cementitious materials, where GGBS fraction in the range of 10–20% is commonly adopted. To ensure comparable flowability across all mixes, the flowability was fixed at 220 mm. All mix proportions were calculated on a dry-mass basis, with the actual mass of SSSM adjusted for its moisture content. Tested properties included flowability, water-to-solid ratio, bleeding rate, compressive strength (3 d, 7 d, 28 d, 60 d), and microstructural analysis (XRD, TG-DTG, SEM). The detailed mix proportions are shown in Table 3.

#### 2.2.1. Raw Material Characterization

The chemical compositions of SSSM, GGBS, and cement were determined using a PANalytical Zetium X-ray fluorescence spectrometer (XRF, Malvern Panalytical B.V., Almelo, The Netherlands). The mineral phase composition of SSSM was analyzed using a PANalytical Empyrean X-ray diffractometer (XRD). The physical properties of SSSM were determined according to the Steel Slag Powder Used for Cement and Concrete (GB/T 20491-2017) [22], and the performance of GGBS was tested according to the Ground Granulated Blast Furnace Slag Used for Cement, Mortar and Concrete (GB/T 18046-2017) [24].

#### 2.2.2. Performance Test Methods

The flowability of CLSM was tested according to the procedures specified in the Technical Standard for Application of Pre-mixed Fluidized Solidified Soil Engineering (DBJ51-T188-2022) [25]. The flowability test was used to evaluate the flow and spreading ability of the fresh slurry. In this study, a target flowability of 220 mm was adopted for all mixes, which is within the recommended range specified by the same standard (180–250 mm), and the water-to-solid ratio of each group was adjusted to achieve a comparable flow state. The water-to-solid ratio was calculated as the ratio of the total water fraction (including both added water and the water carried by SSSM) to the total dry solid mass, according to Equation (1). The bleeding rate was tested in accordance with the Test Method for Free Bleeding Rate and Free Expansion Rate of Cement Paste (T0518-2020) [26], which was used to evaluate the water retention stability of the fresh material slurry during static setting. For compressive strength testing, 70.7 mm × 70.7 mm × 70.7 mm cubic specimens were prepared and cured under standard conditions according to the Standard for Test Method of Basic Properties of Building Mortar (JGJ/T 70-2009) [27]. The compressive strength was measured at 3 d, 7 d, 28 d, and 60 d. For each mix proportion, the average value of three specimens was taken as the test result. All macroscopic performance tests were performed on three parallel specimens for each mix proportion. The results are reported as mean ± standard deviation, and error bars representing standard deviation are added to all macroscopic figures to reflect data variability.

(1) $$w=\frac{m_{1}+m_{2}}{m_{3}},$$

where:

w is the water-to-solid ratio (%);
m_1_ is the water fraction carried by SSSM (g);
m_2_ is the added water (g);
m_3_ is the total mass of dry materials (g).

#### 2.2.3. Simulated Control Test

To verify the reaction mechanism and further elucidate the role of SSSM in the CLSM system, two groups, G9 and G18, were selected for a simulated substitution control test with SSSM. This test did not treat limestone powder, CaSO_4_, and Ca(OH)_2_ as fully equivalent substitutes for SSSM. Instead, inert limestone powder was used to provide a filling matrix, with Ca(OH)_2_ and CaSO_4_ externally added to establish a relatively defined alkali–sulfate activation environment. This setup was designed to compare the strength differences between the alkali–sulfate activation-only condition and the actual SSSM–GGBS system. The dosages of CaSO_4_ and Ca(OH)_2_ were converted on an equivalent basis according to the SO_3_ and Ca(OH)_2_ fractions in SSSM, with the remaining portion supplemented by limestone powder of similar fineness to SSSM. A key assumption of this control test lies in the inert nature of limestone powder under the experimental conditions. Nevertheless, three inherent limitations of this simulation system must be clarified: (1) SSSM contains multiple reactive constituents other than Ca(OH)_2_ and CaSO_4_, which cannot be reproduced in the simulated group; (2) limestone powder fails to completely reproduce the physical nucleation effect of fine SSSM particles on hydration products; and (3) the synergistic interaction between SSSM and GGBS involves complex coupling effects beyond simple alkali–sulfate activation and physical filling. Accordingly, this control test only serves to distinguish extra strength contributions beyond GGBS alkali–sulfate activation within the SSSM–GGBS system, rather than serving as a full equivalent simulation of SSSM hydration behavior. The test results are only suitable for qualitative analysis instead of quantitative calculation. It was not intended as a fully equivalent simulation of the reaction behavior of SSSM. The mix proportions are shown in Table 4 and the strength contribution formula is presented in Equation (2).

(2) $$R_{i}=\frac{f_{si}}{f_{ai}}\times 100\%,$$

where:

R_i_ is the ratio of the simulated control strength to the actual strength at curing age *i* (%);
f_si_ is the compressive strength of the simulated control group at curing age *i* (MPa);
f_ai_ is the compressive strength of the actual SSSM–GGBS group at curing age *i* (MPa);
*i* is the curing age, taken as 3 d, 7 d, 28 d, and 60 d.

**Table 4:** Mix proportions of simulated control groups.

Sample No.	Mass Fraction (wt.%)				
	GGBS	SSSM	CaSO_4_	Ca(OH)_2_	LP
G9	9	91	0	0	0
G9	9	0	1.48	0.99	88.53
G18	18	82	0	0	0
G18	18	0	1.32	0.89	79.79

#### 2.2.4. Sample Preparation for Microstructural Analysis

For microstructural analysis, typical mix proportions were selected for testing. The cemented specimens were cured for 28 d, and after crushing, the central portions were immersed in ethanol for 24 h, followed by replacement of the ethanol and continued immersion for another 48 h to terminate hydration. Subsequently, the samples were dried in an oven at 45 °C for 24 h. The dried samples were then ground to pass through a 0.075 mm sieve. The mineral composition of the samples was determined using PANalytical Empyrean XRD with Cu Kα radiation (λ = 1.5406 Å), operating at 40 kV and 40 mA. Data were collected over a 2θ range of 5° to 80° with a step size of 0.02°. Phase identification was performed using JADE software (https://www.jadeworld.com/). TG-DTG was employed to measure the mass loss of the samples up to 1000 °C at a heating rate of 10 °C/min under a nitrogen atmosphere. Approximately 10 mg of powder sample was placed in an Al_2_O_3_ crucible for each measurement. For morphological observation, the samples were fractured into appropriately sized fragments, and the microstructure was examined using a Zeiss Ultra Plus scanning electron microscope (SEM, Carl Zeiss Microscopy GmbH, Jena, Germany). The specimens were gold-coated (Au) prior to observation to ensure electrical conductivity. The microscope was operated at an accelerating voltage of 5 kV with a secondary electron (SE) detector.

## 3. Results and Discussion

### 3.1. Study on the Influence of GGBS Fraction on the Properties of CLSM

#### 3.1.1. Effect of GGBS Fraction on Water-to-Solid Ratio

**Figure 2:**
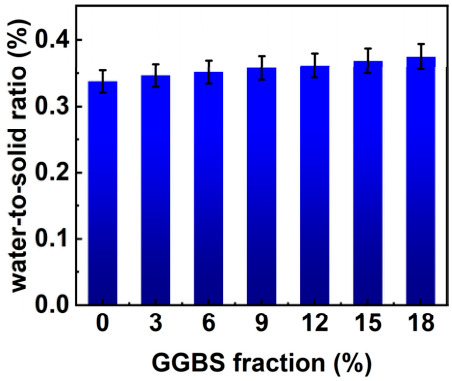
Effect of GGBS fraction on water-to-solid ratio. The ratio increases with GGBS content due to higher water demand.

The effect of GGBS fraction on the water-to-solid ratio of CLSM is shown in Figure 2. At a fixed flowability, the water-to-solid ratio exhibited an increasing trend with an increase in GGBS fraction, rising from 0.337 ± 0.017 at 0% GGBS to 0.375 at 18% GGBS. The incorporation of GGBS required more water to maintain the target flowability. This can be attributed primarily to the higher specific surface area of GGBS particles, which raises the water demand for achieving a given flow state. According to previous studies, a higher GGBS fraction tends to increase the water demand of slurry for a given flow state [28], thus requiring a higher water-to-solid ratio to achieve the specified flowability.

#### 3.1.2. Effect of GGBS Fraction on Bleeding Rate

**Figure 3:**
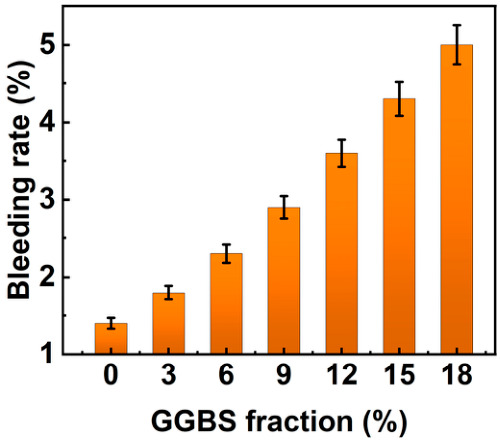
Effect of GGBS fraction on bleeding rate. Bleeding rate increases with GGBS content due to reduced water retention.

The effect of GGBS fraction on the bleeding rate of CLSM is shown in Figure 3. The bleeding rate exhibited an increasing trend with the increase in GGBS fraction, reaching 5 ± 0.25% when the fraction was 18%. This behavior is likely due to the retarding effect of GGBS fraction on early cement hydration: the partial replacement of cement by GGBS retarded the early hydration, which weakened the structural constraint on water migration at early ages and reduced the water retention capacity of the system [28], resulting in an increased bleeding rate.

#### 3.1.3. Effect of GGBS Fraction on the Compressive Strength

**Figure 4:**
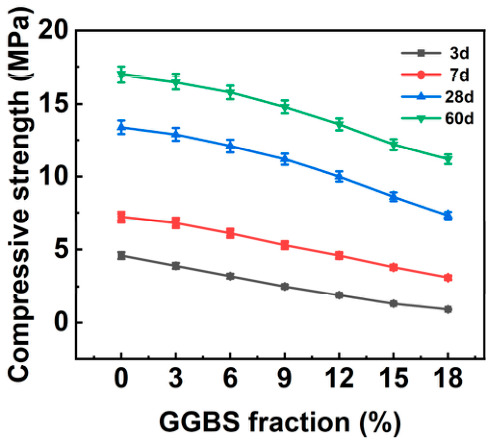
Effect of GGBS fraction on compressive strength. Strength decreases with GGBS content but complete replacement still meets ≤ 8.3 MPa.

The effect of GGBS fraction on the compressive strength of CLSM at various ages is shown in Figure 4. With the increase in GGBS fraction, the compressive strengths of CLSM at all ages exhibited a decreasing trend, which was generally consistent with the fact that the early reaction rate is relatively low and strength development is limited under the low-cement condition with GGBS. Nevertheless, even when cement was completely replaced by GGBS, the 28 d compressive strength still reached 7.3 ± 0.22 MPa, which meets the CLSM requirement of a 28 d compressive strength not exceeding 8.3 MPa. This indicates that the complete replacement of cement by GGBS still satisfies the strength requirements for CLSM, demonstrating the feasibility of using this system as a cementitious component for CLSM.

### 3.2. Study on the Effect of GGBS Fraction on the Performance of SSSM-CLSM

#### 3.2.1. Effect of GGBS Fraction on the Water-to-Solid Ratio of SSSM-CLSM

**Figure 5:**
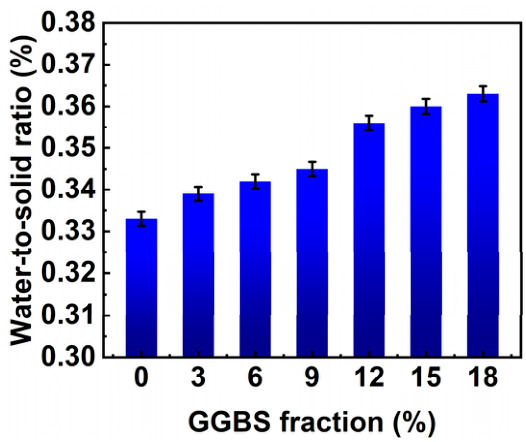
Effect of GGBS fraction on water-to-solid ratio. The trend is consistent with Figure 2.

The effect of GGBS fraction on the water-to-solid ratio of CLSM is shown in Figure 5. As the GGBS fraction increased from 0 to 18%, the water-to-solid ratio of CLSM exhibited an increasing trend, reaching a maximum of 0.363 ± 0.002, which was 9% higher than that of the GGBS-free sample (0.333). This increase can be attributed to the fact that the incorporation of GGBS led to a reduction in the fluidity of the CLSM slurry due to the increased specific surface area of GGBS and its effect on the overall particle packing of the solid mixture; therefore, additional water was required to achieve the specified flowability, resulting in an increased water-to-solid ratio.

#### 3.2.2. Effect of GGBS Fraction on Bleeding Rate of SSSM-CLSM

**Figure 6:**
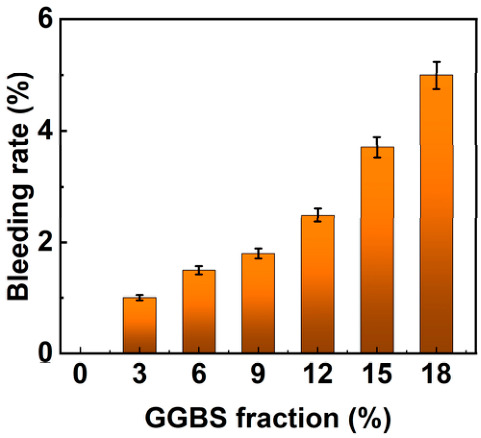
Effect of GGBS fraction on bleeding rate. Bleeding rate increases with GGBS content.

The effect of GGBS fraction on the bleeding rate of CLSM is shown in Figure 6. The bleeding rate increased with GGBS fraction, from 0% at 0% GGBS to 5 ± 0.25% at 18% GGBS. At a GGBS fraction of 0%, the water requirement ratio of SSSM was relatively low, and the free water could be effectively encapsulated. However, after the incorporation of GGBS, the water retention capacity of GGBS was inferior to that of SSSM, and the particle size distribution was altered, reducing stability and increasing the bleeding rate.

#### 3.2.3. Effect of GGBS Fraction on the Compressive Strength of SSSM-CLSM

**Figure 7:**
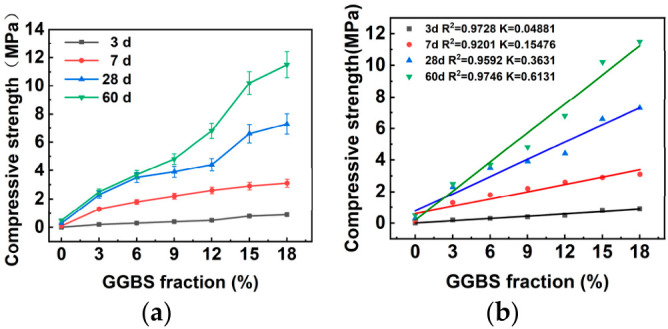
(**a**) Effect of GGBS fraction on compressive strength. (**b**) Fitting curves for the correlation between compressive strength at different curing ages and GGBS fraction.

The effect of GGBS fraction on the compressive strength of CLSM at various ages is shown in Figure 7a. Without GGBS incorporation, the compressive strengths of SSSM-based CLSM at all ages were relatively low. No effective compressive strength was detected at 3 d and 7 d, while the 28 d and 60 d compressive strengths were only 0.3 MPa and 0.5 MPa, respectively, indicating that SSSM itself has poor self-hardening properties. With the increase in GGBS fraction, the compressive strengths of GGBS-CLSM at all ages exhibited an increasing trend. When the GGBS fraction reached 18%, the highest strengths were achieved, with 3 d, 7 d, 28 d, and 60 d compressive strengths reaching 0.9 MPa, 3.1 MPa, 7.3 MPa, and 11.2 MPa, respectively. Compared with the GGBS-free sample, the 3 d and 7 d strengths increased from being undetectable to 0.9 MPa and 3.1 MPa, respectively, while the 28 d and 60 d strengths increased 23.3 and 21.4 times, respectively.

To further quantify this trend, a correlation fitting analysis between strength and GGBS fraction at different ages was conducted, as presented in Figure 7b. The strength of GGBS-based CLSM at each age showed a good linear relationship with the increase in GGBS fraction, with linear correlation coefficients (R^2^) all exceeding 0.90. The slope of the linear fitting curve represents the rate of strength growth. The slopes at 3 d, 7 d, 28 d, and 60 d were 0.04881, 0.15476, 0.3631, and 0.6131, respectively, indicating that the longer the curing age, the greater the slope, and thus the more pronounced the strength increase. It can be seen that increasing the GGBS fraction had a relatively small effect on the 3 d and 7 d strength, but significantly enhanced the later-age strength. By adjusting the GGBS fraction from 0 to 18%, the 28 d compressive strength of CLSM could be regulated within the range of 0.3 MPa to 7.3 MPa, which meets the CLSM strength requirement of 0–8.3 MPa and enables its application in various backfilling operations. It should be noted that the present study mainly focused on the workability and compressive strength of SSSM-based CLSM, while other engineering properties—such as drying shrinkage, durability, freeze–thaw resistance, sulfate resistance, and long-term performance—were not evaluated. Nevertheless, the measured 28 d compressive strength range of 0.3–7.3 MPa covers the typical CLSM requirements for most backfill applications, including trench backfilling and void filling. For applications involving freeze–thaw cycles or sulfate-rich environments, further investigation is required. Future studies will systematically evaluate these engineering properties to facilitate field application.

### 3.3. Solidification Mechanism Analysis of SSSM-CLSM

Both GGBS and SSSM exhibit weak self-hardening properties individually. However, when mixed with water, the CLSM prepared with GGBS and SSSM can achieve a compressive strength of 7.3 MPa. Referring to the hydration mechanisms of the steel slag–GGBS composite cementitious system and the alkali–sulfate synergistically activated GGBS system, hydroxide ions (OH^−^) can promote the dissolution of the glassy structure of GGBS and release reactive components such as Si and Al, while calcium ions (Ca^2+^) and sulfate ions (SO_4_^2−^) further promote the formation of C-S-H gel and ettringite (AFt). These products jointly contribute to strength development through pore filling and skeleton interlocking [29,30,31,32]. Based on this hypothesis, it is proposed that the strength may originate from two aspects: on the one hand, the dissociation of GGBS is believed to occur under the activation of Ca(OH)_2_ carried by SSSM and the hydration reaction involving CaSO_4_, generating cementitious products such as AFt and C-S-H gel; on the other hand, the possible participation of SSSM itself in the reaction may generate additional cementitious products under the alkaline activation induced by GGBS hydration, which may provide a supplementary effect on later-stage densification of the microstructure. To verify the above mechanism and further elucidate the role of SSSM in the CLSM system, a simulated substitution control test with SSSM was conducted in this study. The results of the control test, together with the subsequent XRD, TG-DTG, and SEM analyses, will be presented in the following sections to provide supporting evidence for the proposed mechanism.

**Figure 8:**
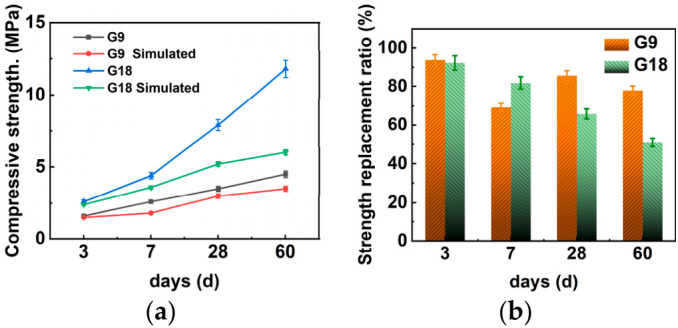
(**a**) Comparison of compressive strength between the actual SSSM–GGBS system and the simulated control group at various ages for G9 and G18 mixes. (**b**) Ratio of the simulated control strength to the actual strength for the corresponding groups.

The compressive strengths at various ages of the simulated control group and the actual group are shown in Figure 8a. It can be seen from Figure 8a that the compressive strengths of the actual SSSM–GGBS system in both G9 and G18 groups were higher than those of the corresponding simulated control groups, indicating that the strength development of CLSM cannot be fully explained solely by the activation of GGBS by Ca(OH)_2_ and CaSO_4_. The fine particle filling effect and potential reactivity of SSSM itself may also exert a certain influence on structural formation and strength development. Further analysis of Figure 8b shows that for the G9 group, at 3 d and 7 d, the strength ratios of the simulated control group to the actual group were 93.75% and 69.23%, respectively, indicating that the early-age strength was similar to that of the alkali–sulfate synergistically activated GGBS system, while the contribution of the potential reactivity of SSSM was relatively limited. At 28 d and 60 d, the strength ratios decreased to 85.71% and 77.78%, corresponding to strength increases in the actual group over the simulated control group of 14.29% and 22.22%, respectively, suggesting that the potentially reactive components in SSSM began to continuously participate in the reaction at later ages and provided a supplementary contribution to strength growth.

In contrast, this difference was more pronounced in the G18 group. At 3 d and 7 d, the strength ratios of the simulated control group to the actual group were 92.31% and 81.82%, indicating that at a high GGBS fraction, the early-age strength still mainly originated from the activated hydration of GGBS under the alkali–sulfate environment, although the potentially reactive components of SSSM had already shown a certain promoting effect. At 28 d and 60 d, the strength ratios decreased significantly to 65.82% and 50.85%, corresponding to strength increases in the actual group over the simulated control group of 34.18% and 49.15%, respectively. These results suggest that under conditions with a higher GGBS fraction, the later-age strength growth of the actual system cannot be attributed solely to the activation of GGBS by Ca(OH)_2_ and CaSO_4_; the secondary reactions of the potentially reactive components in SSSM and the fine particle filling effect made supplementary contributions to later-age strength development.

Based on these findings, the hydration products of the G18 group at 28 d were selected for XRD, TG-DTG, and SEM analyses to further investigate the solidification mechanism.

**Figure 9:**
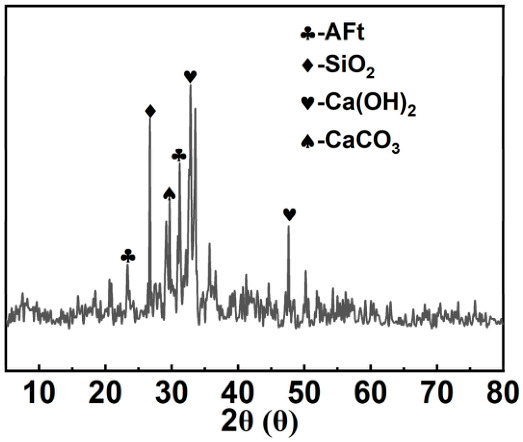
XRD pattern of the G18 group at 28 d. Peaks confirm the formation of AFt, SiO_2_, Ca(OH)_2_, and CaCO_3_.

Figure 9 shows the XRD pattern of the G18 group at 28 d. The diffraction peaks at 2θ = 13.8° and 32.1° indicate the formation of ettringite (AFt); peaks at 2θ = 34.1° and 47.2° correspond to calcium hydroxide (Ca(OH)_2_); and the peak at 2θ = 29.4° is assigned to calcium carbonate (CaCO_3_). Residual unreacted SiO_2_ can also be observed in the pattern. Among these, the presence of AFt indicates that sulfate components in the system participated in the reaction, which is one of the sources of strength. The characteristic peak of Ca(OH)_2_ suggests that a certain alkaline reserve still remained in the system at 28 d. The characteristic peak of CaCO_3_ indicates that a certain degree of carbonation occurred in the specimens. In summary, cementitious products such as AFt had already been formed by 28 d, and a certain alkaline reserve was retained, suggesting that GGBS could be further activated at later ages in the alkali–sulfate environment provided by SSSM. The formation and accumulation of these cementitious products constitute the primary basis for the strength development at 28 d.

It should be noted that the present XRD analysis is qualitative in nature, and no quantitative phase analysis (e.g., Rietveld refinement) was performed. The above discussion regarding the synergistic activation effect between SSSM and GGBS is based on phase identification of the G18 sample combined with existing literature on hydration mechanisms, rather than on quantitative mineral content data. Quantitative phase analysis will be carried out in future work to further elucidate the variation in hydration product content.

**Figure 10:**
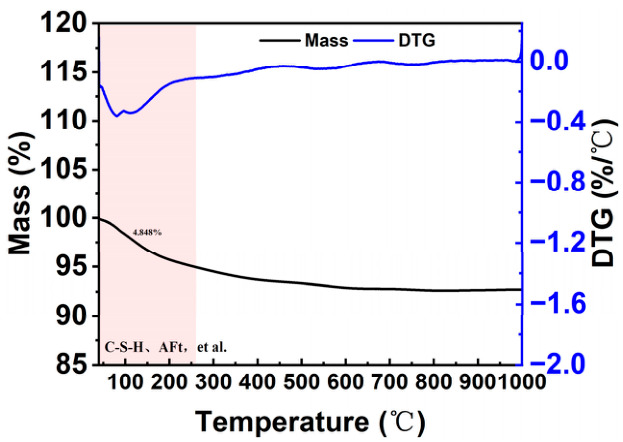
TG-DTG curves of the G18 group at 28 d. The mass loss of 4.848% at 50–250 °C exceeds the theoretical AFt crystalline water loss (2.84%), confirming substantial C-S-H gel formation alongside AFt.

Figure 10 shows the TG-DTG curves of the G18 group at 28 d. As can be seen from Figure 10, the main mass loss region of the G18 group is between 50 and 250 °C, which mainly corresponds to the dehydration of C-S-H gel and AFt. Based on the G18 mix proportion and the chemical composition of the raw materials, it can be calculated that the SO_3_ fraction in the system is 1.184%. If all SO_3_ in the system participated in the formation of AFt, the generated AFt fraction would be 6.19%, corresponding to a crystalline water loss of 2.84%. The TG-DTG results show that the mass loss of the G18 group is 4.848%, significantly higher than the combined water loss of AFt, indicating that the mass loss in this region includes not only the crystalline water of AFt but also the dehydration of a considerable amount of C-S-H gel. Furthermore, assuming that f-CaO in SSSM serves as the direct calcium source for AFt formation, the generated AFt would be 2.63%, with a corresponding combined water loss of only 1.21%, which is markedly lower than the total mass loss. This indirectly suggests that the formation of AFt in the system also relies on the participation of other calcium-containing mineral phases in SSSM and the calcium source released during the activation process of GGBS. This implies that the formation of AFt does not simply originate from the hydration of f-CaO, and is more likely the result of a synergistic reaction between GGBS and SSSM. Combined with the characteristic peaks of AFt, Ca(OH)_2_, and CaCO_3_ detected by XRD, the strength development of the G18 group at 28 d can be primarily attributed to the sustained reaction of GGBS under the alkali–sulfate environment, generating cementitious products such as AFt and C-S-H gel. In this process, SSSM not only provides the alkaline and sulfate environment required for activation but also participates in the synergistic solidification process through its calcium-containing and potentially reactive components.

**Figure 11:**
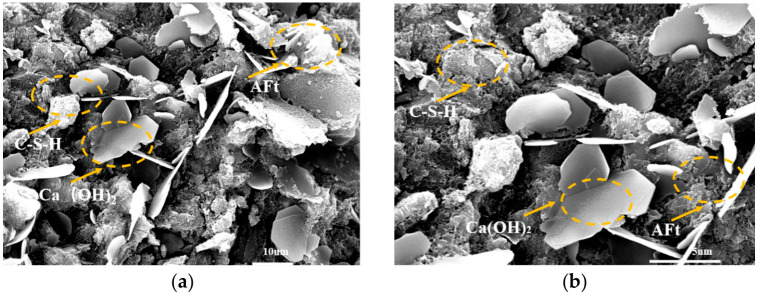
SEM images of the G18 group at 28 d. A dense microstructure with flocculent C-S-H gel and needle-like AFt is observed: (**a**) 3000×; (**b**) 5000×.

Figure 11 shows the SEM images of the G18 group at 28 d. As can be seen from Figure 11, at 28 d, a large amount of flocculent or clustered C-S-H gel, a small amount of needle-like or short rod-like AFt, and flaky Ca(OH)_2_ can be observed inside the specimens. AFt and C-S-H gel intertwined and grew together, forming a more complete spatial cementitious structure. Meanwhile, the interfaces between particles were further encapsulated and connected by the newly formed solidification products, and the pore size and pore connectivity were significantly reduced, indicating that a relatively continuous cementitious network had been formed in the specimens at 28 d. This structural feature suggests that the cementitious products generated from the combination of GGBS and SSSM through pore filling, enhancement of interparticle cementation, and improvement of interfacial bonding gradually promoted the evolution of the specimens into a dense structure, offering microstructural evidence that is consistent with the observed mechanical performance.

## 4. Conclusions

In this study, SSSM was used as the base material, with cement and GGBS as cementitious materials, to prepare SSSM-based CLSM. A two-stage experimental program was designed: GGBS first replaced cement (0–18%), and then, based on the optimized mix, GGBS replaced SSSM (0–18%). The simulated control test, using limestone powder, Ca(OH)_2_, and CaSO_4_, was conducted to distinguish the contributions of alkali–sulfate activation and the intrinsic effects of SSSM.

1. Increasing the GGBS fraction increased the water-to-solid ratio and bleeding rate but decreased compressive strength. When cement was completely replaced by GGBS, the 28 d compressive strength still reached 7.3 MPa, meeting CLSM requirements. The linear fitting of strength–GGBS fraction showed R^2^ > 0.90, with higher slopes at later ages, indicating more pronounced strength enhancement over time.
2. The simulated control test showed that the actual SSSM–GGBS system outperformed the simulated control at all ages. For the G18 group, the actual-to-control strength ratios decreased from 92.31% (3 d) to 50.85% (60 d), meaning the actual system exceeded the control by 49.15% at 60 d. The observation suggests that SSSM contributes not only through alkali–sulfate activation but also via fine particle filling and secondary reactions of its potentially reactive components.
3. Microstructural analyses (XRD, TG-DTG, SEM) indicated the formation of AFt and C-S-H gel as the primary products. TG-DTG showed a mass loss of 4.848% at 50–250 °C, higher than the theoretical AFt crystalline water loss (2.84%), indicating substantial C-S-H formation. AFt formation required calcium sources beyond f-CaO, suggesting the occurrence of synergistic reactions between GGBS and SSSM. SEM revealed a dense cementitious network with flocculent C-S-H gel and needle-like AFt, supporting the 7.3 MPa strength.

Overall, this work shows that SSSM can effectively be matched with fully cement-substituted GGBS to fabricate CLSM satisfying the strength requirements for CLSM backfill applications. The combination of macroscopic mechanical tests, simulated control tests, and multi-scale microstructural analyses (XRD, TG-DTG, SEM) suggests the dual role of SSSM: providing an alkali–sulfate activation environment for GGBS hydration, while also contributing to strength development through the reactivity of its mineral components and microaggregate filling effects. Future work will focus on evaluating key engineering properties (e.g., setting time, drying shrinkage) and long-term environmental performance to facilitate the field application of this solid waste-based CLSM.

## Figures and Tables

**Table 1:** Chemical composition of SSSM, GGBS, and cement (wt.%).

Material	Mass Fraction/%										
	LOI	CaO	SiO_2_	Fe_2_O_3_	MgO	Al_2_O_3_	MnO	Cr_2_O_3_	Na_2_O	SO_3_	Σ
SSSM	1.90	45.98	28.09	7.22	4.74	3.39	1.08	3.04	0.18	0.99	98.50
GGBS	1.04	40.00	30.04	0.29	10.66	14.92	-	-	0.41	2.07	99.43
Cement	0.38	65.49	21.21	3.50	1.64	5.84	-	-	-	-	98.06

**Table 2:** Heavy metal leaching concentration of SSSM.

Element	As	Hg	Be	Cd	Cr	Ni	Pb	Cr(VI)	Ag
Leaching concentration (mg/L)	0.5	0.05	0.005	0.1	1.5	1.0	1.0	0.5	0.5
Limit value (mg/L)	0.0015	0.0002	<0.0007	<0.0012	0.388	0.404	<0.0042	0.2	<0.0029

**Table 3:** Mix proportion for experiment (wt.%).

Sample No.	Mass Fraction (wt.%)		
	Cement	GGBS	SSSM
C18	18	0	82
C15G3	15	3	82
C12G6	12	6	82
C9G9	9	9	82
C6G12	6	12	82
C3G15	3	15	82
G18	0	18	82
G0	0	0	100
G3	0	3	97
G6	0	6	94
G9	0	9	91
G12	0	12	88
G15	0	15	85

C18–G18: composite binder series with GGBS partially or fully replacing cement (0–18%). C15G3: 15% cement + 3% GGBS; G18: 18% GGBS, fully replacing cement. G0–G18: series based on the optimized G18 formula from the C18–G18 group, with GGBS replacing SSSM at mass fractions of 0–18% (e.g., G6: 6% GGBS replacing SSSM).

## Data Availability

The original contributions presented in this study are included in the article. Further inquiries can be directed to the corresponding author.
